# Efficacy and safety of ketamine for neonatal refractory status epilepticus: case report and systematic review

**DOI:** 10.3389/fped.2023.1189478

**Published:** 2023-06-02

**Authors:** Jacopo Norberto Pin, Letizia Leonardi, Margherita Nosadini, Maria Elena Cavicchiolo, Chiara Guariento, Anna Zarpellon, Giorgio Perilongo, Alessia Raffagnato, Irene Toldo, Eugenio Baraldi, Stefano Sartori

**Affiliations:** ^1^ Department of Women’s and Children’s Health, Paediatric Neurology and Neurophysiology Unit, University Hospital of Padua, Padova, Italy; ^2^ Master in Pediatrics and Pediatric Subspecialties, University Hospital of Padua, Padova, Italy; ^3^ Neuroimmunology Group, Paediatric Research Institute “Città della Speranza”, Padova, Italy; ^4^ Department of Women’s and Children’s Health, Neonatal Intensive Care Unit, University Hospital of Padua, Padova, Italy; ^5^ Department of Women’s and Children’s Health, Child and Adolescent Neuropsychiatric Unit, University Hospital of Padua, Padova, Italy; ^6^ Department of Neuroscience, University Hospital of Padua, Padova, Italy

**Keywords:** ketamine, refractory status epilepctius (RSE), neonatal status epilepticus, neonatal seizure treatment, antiseizure medication response

## Abstract

**Background:**

Evidence-based data on treatment of neonatal status epilepticus (SE) are scarce. We aimed to collect data on the efficacy and safety of ketamine for the treatment of neonatal SE and to assess its possible role in the treatment of neonatal SE.

**Methods:**

We described a novel case and conducted a systematic literature review on neonatal SE treated with ketamine. The search was carried out in Pubmed, Cochrane, Clinical Trial Gov, Scopus and Web of Science.

**Results:**

Seven published cases of neonatal SE treated with ketamine were identified and analyzed together with our novel case. Seizures typically presented during the first 24 h of life (6/8). Seizures were resistant to a mean of five antiseizure medications. Ketamine, a NMDA receptor antagonist, appeared to be safe and effective in all neonates treated. Neurologic sequelae including hypotonia and spasticity were reported for 4/5 of the surviving children (5/8). 3/5 of them were seizure free at 1–17 months of life.

**Discussion:**

Neonatal brain is more susceptible to seizures due to a shift towards increased excitation because of a paradoxical excitatory effect of GABA, a greater density of NMDA receptors and higher extracellular concentrations of glutamate. Status epilepticus and neonatal encephalopathy could further enhance these mechanisms, providing a rationale for the use of ketamine in this setting.

**Conclusions:**

Ketamine in the treatment of neonatal SE showed a promising efficacy and safety profile. However, further in-depth studies and clinical trials on larger populations are needed.

## Introduction

Status epilepticus (SE) is defined as a condition resulting either from the failure of the mechanisms responsible for seizure termination or from the initiation of mechanisms which lead to abnormally prolonged seizures (after time point t1, which in the case of convulsive SE is at 5 min). SE can have long-term consequences (after time point t2, that for convulsive SE is after 30 min), including neuronal death, neuronal injury, and alteration of neuronal networks, depending on seizure type and duration (1). Status epilepticus that persists despite administration of at least 2 appropriately dosed antiseizure medications including a benzodiazepine is referred to as refractory status epilepticus (RSE) (2).

The definition of SE used in adults is only partially applicable to neonates; a widely accepted definition describes neonatal SE as a seizure lasting thirty minutes or a series of seizures whose total duration exceeds 50% of electroencephalography (EEG) record (3, 4). Neonatal seizures are a common emergency with an incidence that ranges between 0.95–3.5/1,000 for term newborns and 10–130/1,000 live births for preterms (5–7). SE is reported in 8%–43% of newborns with seizures (8–10). The main aetiologies of neonatal seizures are fully described in Supplementary Table S1 (6, 11). It is debated if all neonatal seizures should be treated due to the potential side effects of antiseizure medications (ASMs). Besides, in the absence of evidence-based studies on neonatal population, numerous attempts are made to use ASMs whose efficacy has already been established in adults (12–14).

In this respect, there is increasing interest towards the use of ketamine. Little is known regarding dosing, safety, and efficacy for the treatment of neonatal SE and its use is still rare.

The aim of this study was to report the case of a newborn with hypoxic-ischemic encephalopathy (HIE) who developed a RSE successfully treated with ketamine and to review the literature, providing an up-to-date summary of the available evidence on the use of ketamine for the treatment of neonatal RSE.

## Materials and methods

We described a case of RSE successfully treated with ketamine in a newborn with HIE.

**Figure 1:**
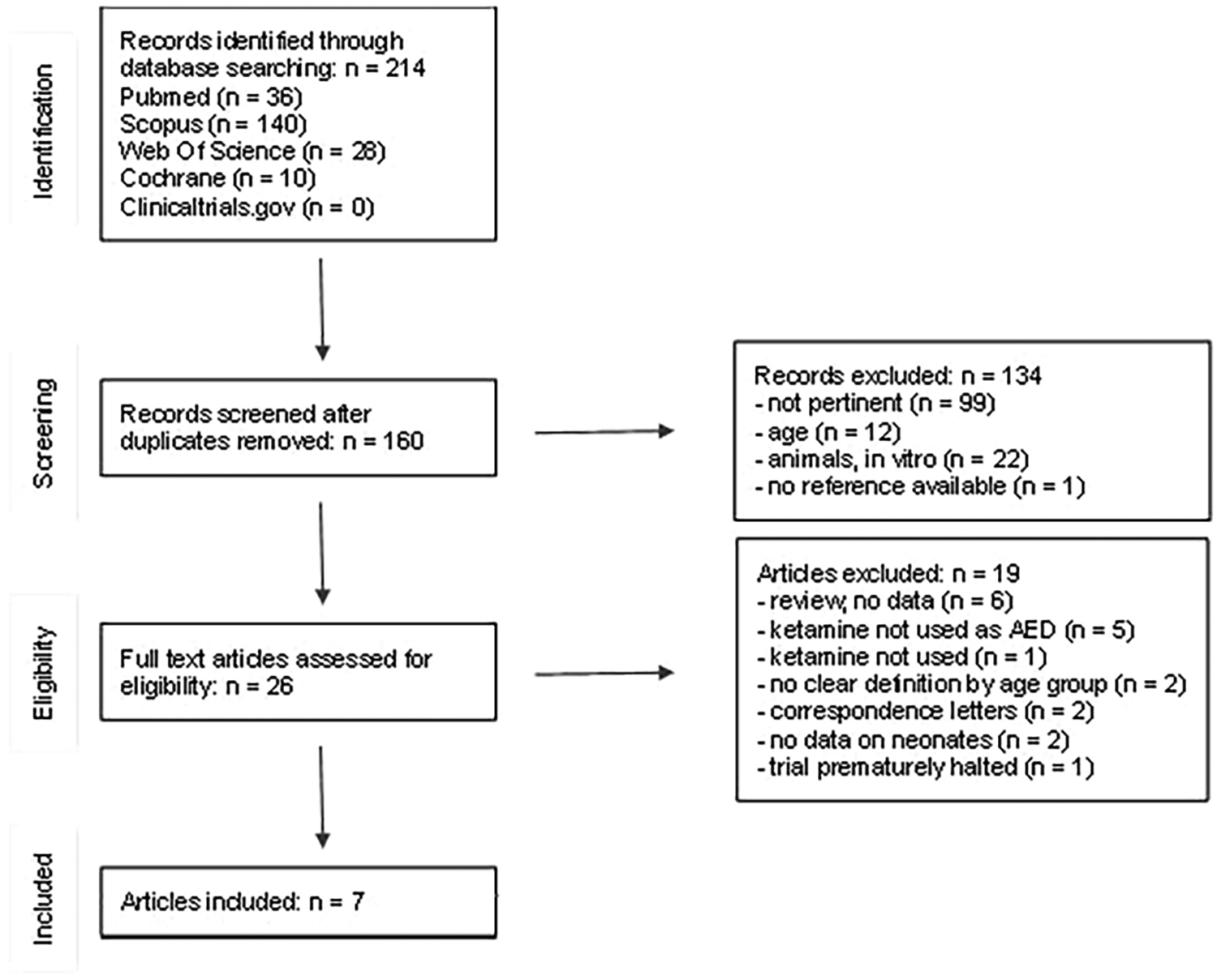
Identification of studies.

We then performed a systematic literature review on the use of ketamine in neonatal status epilepticus. The search was carried out in Pubmed, Cochrane, Clinical Trial Gov, Scopus and Web of Science by two researchers (LL and JNP), up-to-date to December 2022 (Figure 1), complying with the PRISMA guidelines.

The search terms used were (((newborn) AND (seizure)) AND (ketamine)), (((newborn) AND (status epilepticus)) AND (ketamine)), ((neonatal seizure) AND (ketamine)). We also considered clinicaltrial.gov for ongoing trials. No language restrictions were applied. The available articles were filtered manually for patients in neonatal age (≤1 month of life) with SE treated with ketamine. Demographics, clinical, diagnostic and treatment data were collected. Studies with mixed population (neonates, children and adults) without a clear division by age groups, or reviews not reporting data on new patients, were excluded. Studies in which ketamine was not used as an ASM were excluded.

## Results

### Case report

A male infant was born at 40 + 3/7 gestational weeks, after an uneventful pregnancy. There was no relevant family history. Labor and vaginal delivery were uncomplicated, except for meconium-stained amniotic fluid. Birth weight, head circumference and length were all appropriate for gestational age. At birth, he was hypotonic, without spontaneous respiratory activity. Heart rate was stably >100 beats per minute. T-piece ventilation was immediately started followed by nasotracheal intubation at 10 min of life, with subsequent rapid improvement of activity and tone. At 19 min of life the newborn was extubated, showing good spontaneous respiratory activity and blood saturation values. Apgar scores were 6, 7, 7 and 8 at 1′, 5′, 10′ and 15′, respectively. Venous cord blood pH was 7.30, pCO2 31.3 mmHg and base deficit of −11.8 mmol/L. At 1 h of life, arterial blood gas analysis showed metabolic acidosis (pH 7.03, HCO3 13.8 mmol/L, BE −16.9 mmol/L), but since Sarnat and Sarnat score was 0, therapeutic hypothermia was not started. At 18 h of life, the baby presented apnoeic episodes with desaturation followed by bilateral clonic jerks. He was transferred to a hub hospital, where continuous electroencephalography (EEG) recording revealed multifocal seizures. At 36 h of life cerebral magnetic resonance imaging (MRI) showed abnormal diffusion restriction in fronto-temporal-parietal cortical/subcortical regions and thalamus bilaterally. Metabolic and genetic (array-comparative genomic hybridization (aCGH) and epilepsy comprehensive panel were performed) aetiologies were investigated and excluded. The boy soon developed a refractory status epilepticus (RSE): seizures were resistant to phenobarbital (max dose 40 mg/kg/die), levetiracetam (70 mg/kg/die), phenytoin (10 mg/kg/die), midazolam (1 mg/kg/h), lidocaine (7 mg/kg/h, for 36 h) and vitamin B6 (100 mg/kg bolus, then 15 mg/kg/die). Thiopental (5 mg/kg/h) made it possible to control clinical epileptic seizures, with persistence of electrical seizures. At the age of 8 days, he was referred to our neonatal intensive care unit. With informed consent, he was started on ketamine at 10 mcg/kg/min, with continued EEG and vital signs monitoring. Ketamine was titrated up to 100 mcg/kg/min in order to control the electro-clinical seizures. It was therefore possible to gradually discontinue all other ASMs and sedative drugs. Ketamine was administered for a total of 8 days. No side effects were reported. At 2 weeks of life, cerebral MRI showed diffuse white matter oedema with concomitant bilateral necrotic evolution of previously found lesions. Proton spectroscopy study of the right nucleocapsular region showed an elevated lactate peak consistent with hypoxic-ischemic injury.

Due to severe neurological impairment, extensive brain damage and absence of spontaneous respiratory activity, a palliative care pathway was started in agreement with the parents. The baby died at 23 days of life.

### Literature review

The literature search yielded a total of 214 articles (Figure 1). 54 records were excluded because they were duplicates. 133 additional records were excluded after title and abstract examination: 12 studies including only adults or children older than 1 month, 99 studies not related to neonatal SE, 22 studies made on animals or *in vitro*. A total of 27 full-text articles were assessed for eligibility. After full-text examination, 20 more studies were excluded: 6 reviews not reporting raw data (5, 11, 15–18), 2 articles not reporting data on neonates (19, 20), 2 studies without a clear division by age groups (21, 22), 1 article in which ketamine was not mentioned (23). Ketamine was not used as ASM in 5 studies (24–28); 1 trial that was prematurely halted (29) and 2 correspondence letters (over one of the case reports already included) were excluded (30, 31). A final number of 7 studies were included (32–38), (Figure 1). The articles were analyzed and described below and in Table 1. We also considered our case report, for a total of 8 neonatal cases included. Of the neonates included, six out of eight were born at term, one was born at 34 gestational weeks (35), and in one case gestational age was not reported (32). We did not notice a gender prevalence. Six out of eight neonates were born from uncomplicated, uneventful pregnancies. In one case prenatal diagnosis of Pierre-Robin syndrome associated with lissencephaly and polymicrogyria was made. For one case data were not available (32). Perinatal history of respiratory failure and/or hypotonia was present in 6/7 cases. In one case perinatal history was not available (38). Onset of symptoms occurred during the first 24 h of life (6/8), except for two cases, whose first symptoms started within the third day of life. At clinical presentation signs and symptoms included seizures (8/8), apnoea and desaturation (6/8). EEG was performed in all cases. Burst suppression pattern was the most frequent finding at presentation (5/8), often associated with multifocal seizures (3/8).

**Table 1:** Summary of evidences.

	Demographic			Diagnosis				Antiseizure medications								Outcomes		
	Sex	Gestational age (weeks)	5 min Apgar	Onset symptoms	Age at presentation (days)	Brain imaging	Diagnosis	1st line	2nd line	3rd line	4th line	5th line	6th line	7th line	8th line	Efficacy of Ketamine	Side effects	Death
Pin 2022	Male	40 + 3	7	Apnea, desaturation, seizures	1	MRI: abnormal diffusion restriction bilaterally in cortical/subcortical regions and thalamus	Hypoxic-ischemic encephalopathy	Phenobarbital	Levetiracetam	Phenytoin	Midazolam infusion	Lidocaine	Vitamin B6	Thiopental	Ketamine	Yes, cessation	No	Yes (23 days)
Mandal 2022	Female			Seizures	1	MRI: partial parieto- occipital hemimegalencephaly	Partial hemi- megalencephaly	Phenobarbital	Levetiracetam	Phenytoin	Midazolam infusion	Lacosamide	Ketamine	Topiramate		Yes, reduction	No	No
Huntsman 2019	Male	39 + 1	5	Desaturation, seizures	1	MRI: large subacute infarction and hemorrhages	Hypoxic-ischemic encephalopathy and hemorrhagic stroke	Phenobarbital	Phenytoin	Midazolam infusion	Topiramate	Ketamine				Yes, cessation	No	No
Freibauer 2018	Male	38	4	Apnea, desaturation, seizures	1	MRI: patchy areas of hyperintense T2 signal in the deep white matter	KCNQ2 related encephalopathy	Phenobarbital	Phenytoin	Midazolam	Ketamine					Yes, cessation	No	Yes (37 days)
Tarocco 2014	Female	34		Apnea, seizures	1		Epileptic encephalopathy, lissencephaly and polymicrogyria	Phenobarbital	Phenytoin	Midazolam	]Levetiracetam	Propofol	Ketamine	Carbamazepine		Yes, cessation	No	Yes (103 days)
Dhamija 2012		41 + 5	9	Apnea, seizures	2	MRI: corpus callosum agenesis; immature sulcation pattern; spectroscopy with elevated glycine peak	Non ketotic hyperglycinemia encephalopathy	Phenobarbital	Ketamine							Yes, cessation	No	No
Tegtmeyer 1995	Female	40	10	Seizures	1		Non ketotic hyperglycinemia encephalopathy	Diazepam	Midazolam	Ketamine						Yes, cessation	No	No
Haider 1996	Male	Term	9	Apnea, lethargy, seizures	3	CT: low attenuated areas in fronto-parietal regions	Non ketotic hyperglycinemia encephalopathy	Phenobarbital	Phenytoin	Ketamine						Yes, cessation	No	No

Phenobarbital was used as the first ASM in all except for one case that was initially treated with diazepam. In 4/8, phenytoin represented the second line treatment, usually followed by midazolam infusion or administration. In 2/8 cases levetiracetam was administered as second line treatment before phenytoin, followed by midazolam infusion as fourth line of treatment in both cases. Ketamine was chosen as second line treatment only in one case, not followed by administration of any other drugs (36), whereas it was used as the third or subsequent therapeutic line in the remaining 7/8 cases. Ketamine dosage ranged from 1.5 mcg/kg/h up to 100 mcg/kg/h and was administered for a total of 8–28 days. Ketamine was effective in cessation (7/8) or reduction (1/8) of seizures, inducing control of SE in all cases included. No acute side effects related to the use of ketamine were reported. Three neonates died at a mean age of 54±34 days of life. After initial control of seizures, in two cases it was decided to withdraw care due to the overall poor prognosis and the patients died at 37 and 103 days of life, respectively (34, 35). In one patient, status epilepticus reappeared after 15 days free of seizures (35). At follow-up (mean time 3–17 months), neurological outcomes of the remaining cases (5/8) mainly included hypotonia and poor sucking (4/5) or spasticity (3/5). In one case functional hemispherectomy led to definitive seizures cessation at 14 months of life (32). Three other cases were reported to have achieved complete seizure cessation by the age of 3–17 months (33, 37, 38). Only in one case poor control of seizures was documented (36).

## Discussion

In this study we aimed to investigate the efficacy and safety profile of ketamine for the treatment of neonatal SE. As regards the general characteristics of the cases included, neonates were typically born from uncomplicated pregnancies. Despite this, in most cases perinatal history of hypotonia or respiratory failure at birth was documented, most likely due to a hypoxic injury at the moment of delivery. All patients presented with seizures, whose onset was during the first 24 h of life in 75% of cases. Children whose onset of symptoms started during the next days of life were the only ones reported to be healthy at birth. We did not notice a prevalence of pre-term neonates. Timing of presentation and the absence of clear previous risk factors are in most of included cases suggestive of neonatal encephalopathy, especially of HIE (39, 40). The gold standard for diagnosis of SE is EEG, which was performed in all cases immediately after the onset of seizures (2, 41). Recent guidelines have emphasized that EEG is required for identification, diagnosis and confirmation of neonatal seizures, including conventional EEG (cEEG) and amplitude-integrated EEG (aEEG) (2, 42). In contrast to this, with clinical observation alone only focal clonic and focal tonic seizures can be diagnosed if observed by an expert (2, 41). In most of our cases burst-suppression pattern was found, consistent with a hypoxic injury (43).

Currently, the most common first-line medication indicated for SE in the neonate is phenobarbital (14, 44–46). Accordingly, phenobarbital was used as the first ASM in 88% of cases, usually followed by the administration of phenytoin, midazolam or levetiracetam. Existing evidence suggests initiation with phenobarbital at the dosage of 20 mg/kg (3, 42). Neonates with persistence of seizures may receive an additional 20 mg/kg of phenobarbital (45). The only neonate that received ketamine as a second therapeutic line was affected by NKH, where ketamine is often used due to its antagonist action on the NMDARs, which are hyperactivated by the high levels of glycine. Glycine is a co-agonist of NMDAR, together with glutamate (23). Its excess is thought to account for the onset of seizures in neonates with NKH, but also for their long-term neurological sequelae (20, 47, 48).

In all other cases (88%), ketamine was used as a third or subsequent line of treatment. It was effective in controlling RSE in all cases included. The width of the range of ketamine doses used (1.5–100 mcg/kg/h) reflects the absence of any recommendation on this topic.

SE is particularly difficult to treat in neonates: rapid neurodevelopmental mechanisms that take place in the neonatal brain result in a lower threshold for seizures. Immature neonatal brain presents a shift in the action of gamma-aminobutyric acid (GABA) from inhibition to excitation, and a greater density of NMDAR subunits, which promote prolonged excitatory postsynaptic potentials (5, 33, 49–51). In addition to this, extracellular concentrations of glutamate are higher in neonates due to increased release and decreased reuptake at the level of the synaptic cleft (52, 53).

In neonates, the balance between excitatory and inhibitory forces can be additionally altered by cerebral insults such as HIE or prolonged SE itself. In fact, they all induce an upregulation and/or hyperactivation of NMDARs, an increase of glutamate expression, and a reduction in post-synaptic GABA receptors, further promoting the persistence of seizures (52, 54).

Ketamine is a non-competitive NMDA glutamate receptor antagonist. NMDAR is a non-specific transmembrane cation channel made of five subunits that form a ligand and voltage gated channel. Activation of NMDAR on the post-synaptic neuronal membrane induces an influx of sodium and calcium ions that in the end results in an excitatory potential. Ketamine acts on the phencyclidine binding site present within the NMDAR channel, blocking this cations influx, and exerting its NMDAR antagonist activity (55).

In the mature brain, activation of GABA determines hyperpolarization of neurons thanks to a net influx of Cl^−^ anions, resulting in an inhibitory effect (56). In neuroblasts and immature neurons, the presence of an inwardly directed Na^+^-K^+^-Cl^−^ co-transporter 1 (NKCC1) determines a relatively high internal Cl^−^ concentration. Thus, GABA receptor activation results in a net efflux of anion and cell depolarization, and explains the paradoxical excitatory effects of GABA, seen in pre-term neonates (58). Progressive acquisition of NKCC2 co-transporter on neurons decreases the internal chloride concentration, restoring the inhibitory GABA activity (54, 57–59).

Moreover, persistence of seizures determines the internalization inside the cell of GABA receptors that remain pharmacologically responsive only to very high doses of neurosteroids (allopregnanolone, allotetrahydrodeoxycorticosterone) or midazolam (60).

These mechanisms could explain the reduced efficacy of GABAergic ASMs, especially in pre-term neonates. It has been highlighted that despite correct initial loading doses of standard ASMs, more than half of neonates experience continued seizures (61). Key challenges in the management of this condition surround the incomplete efficacy of ASM and the concomitant higher rate of side effects with increasing dosage of GABAergic medications. In this setting, ketamine represents a viable therapeutic option in the treatment of neonatal RSE. Given the high rate of neurological sequelae expected after neonatal SE, which in older children happen in more than 50% of cases, rapid cessation of seizures should be pursued (62, 63). After administration of ketamine, in the acute phase there was control of seizures in all cases included. However, in two cases there was recurrence of seizures (34, 35). After a mean duration of therapy of 17.2 days, no side effects were highlighted. In contrast to this latter point, high doses of GABAergic ASMs often determine acute negative cardiovascular effects, in particular hypotension. Ketamine, owing to its sympathomimetic effects, does not necessarily require mechanical ventilation or amine administration, which have been identified as negative prognostic factors both in adults and children (17, 29, 64–67). **However, we suggest continuous monitoring of vital signs in association with EEG, since this allows better characterization of seizures and differentiation from non-epileptic paroxysmal events. Also, it permits prompt identification of any possible side effects that could arise during ketamine infusion**(45)**.**

At 3–17 months, it was reported good seizure control in 80% of the surviving patients; however, they presented with predominantly motor sequelae (such as hypotonia or spasticity) (32, 33, 36–38). There is no consensus over the long-term consequences of ketamine administration in neonates. Studies on adults have shown that prolonged ketamine use may lead to neuropsychiatric impairment, urologic or hepato-biliary injury (68–70). Ketamine is metabolized in the liver by the CYP3A4 system, then excreted through the kidneys and, to a lesser extent, the biliary system. Involvement of both the urinary and biliary tracts suggests that ketamine or its metabolite (norketamine) may have direct toxic effect on the urinary/biliary tract epithelium (71). Following ketamine administration in rats and nonhuman primates, upregulation of NMDARs has been observed, which after its dismissal would lead to neuronal degeneration and apoptosis (35, 72–75).

Mortality rate in neonatal SE is reported to be around 9%–24% (76, 77). In our review the overall mortality rate was 37.5%. This higher rate may be related to the small sample size of our cohort, but also to the severity of the underlying pathology of the neonates affected, as in our case report.

Limitations to this study included the very small population and the heterogeneity of data collected from the selected articles, which did not allow to perform a metanalysis. In some cases, it was not possible to ascertain the raw data.

## Conclusions

To the best of our knowledge this is the first systematic review on the use of ketamine for the treatment of neonatal SE. **Evidence suggests that ketamine could be used as third line treatment, due to its pharmacodynamic, different from the most used GABA-ergic ASM.**

Despite the above-mentioned limitations, ketamine showed a promising efficacy and safety profile. Rationale for its use in this setting finds confirmation both in pharmacodynamics mechanisms and in the peculiar anatomic-functional characteristics of neonatal brain. Given the pathophysiology of the immature brain, its efficacy could be even higher in newborns, highlighting its possible role in terms of precision medicine for the treatment of neonatal SE. However, our study is limited by a knowledge gap in the long-term outcomes of ketamine administration in neonates, that yet needs to be properly assessed in randomized clinical trials and larger studies.

## References

[1] Trinka E Cock H Hesdorffer D Rossetti AO Scheffer IE Shinnar S A definition and classification of status epilepticus–report of the ILAE task force on classification of Status epilepticus. Epilepsia. (2015) 56(10):1515–23. 10.1111/epi13121 26336950

[2] Hirsch LJ Gaspard N van Baalen A Nabbout R Demeret S Loddenkemper T Proposed consensus definitions for new-onset refractory Status epilepticus (NORSE), febrile infection-related epilepsy syndrome (FIRES), and related conditions. Epilepsia. (2018) 59(4):739–44. 10.1111/epi.14016 29399791

[3] Pressler RM Cilio MR Mizrahi EM Moshé SL Nunes ML Plouin P The ILAE classification of seizures and the epilepsies: modification for seizures in the neonate. Position paper by the ILAE task force on neonatal seizures. Epilepsia. (2021) 62(3):615–28. 10.1111/epi.16815 33522601

[4] Abend NS Wusthoff CJ. Neonatal seizures and status epilepticus. J Clin Neurophysiol. (2012) 29(5):441–8. 10.1097/WNP.0b013e31826bd90d 23027101 PMC3463810

[5] Ziobro JM Eschbach K Shellhaas RA Novel therapeutics for neonatal seizures. Neurotherapeutics (2021) 18(3):1564–81. 10.1007/s13311-021-01085-8 34386906 PMC8608938

[6] Padiyar S Nusairat L Kadri A Abu-Shaweesh J Aly H. Neonatal seizures in the U.S. National inpatient population: prevalence and outcomes. Pediatr Neonatol. (2020) 61(3):300–5. 10.1016/j.pedneo.2019.12.006 31937508

[7] Baudou E Cances C Dimeglio C Hachon Lecamus C. Etiology of neonatal seizures and maintenance therapy use: a 10-year retrospective study at Toulouse children’s hospital. BMC Pediatr. (2019) 19(1):136. 10.1186/s12887-019-1508-5 31035972 PMC6487521

[8] Wertheim D Mercuri E Faundez JC Rutherford M Acolet D Dubowitz L. Prognostic value of continuous electroencephalographic recording in full term infants with hypoxic ischaemic encephalopathy. Arch Dis Child Fetal Neonatal Ed. (1994) 71(2):F97–102. 10.1136/fn.71.2.f97 7979486 PMC1061091

[9] McBride MC Laroia N Guillet R. Electrographic seizures in neonates correlate with poor neurodevelopmental outcome. Neurology. (2000) 55(4):506–14. 10.1212/WNL.55.4.506 10953181

[10] Pavlidis E Spagnoli C Pelosi A Mazzotta S Pisani F. Neonatal Status epilepticus: differences between preterm and term newborns. Eur J Paediatr Neurol. (2015) 19(3):314–9. 10.1016/j.ejpn.2015.01.002 25613545

[11] Ahrens S Ream MA Slaughter LA. Status epilepticus in the neonate: updates in treatment strategies. Curr Treat Options Neurol. (2019) 21(2):8. 10.1007/s11940-019-0546-5 30773607

[12] Heuser K Olsen KB Ulvin LB Gjerstad L Taubol E. Modern treatment of status epilepticus in adults. In: Czuczwar SJ, editors. Epilepsy. Brisbane, AU: Exon Publications (2022). 35605086

[13] Gaspard N Foreman BP Alvarez V Cabrera Kang C Probasco JC Jongeling AC Critical care EEG monitoring research Consortium (CCEMRC). New-onset refractory status epilepticus: etiology, clinical features, and outcome. Neurology. (2015) 85(18):1604–13. 10.1212/WNL.0000000000001940 26296517 PMC4642147

[14] Alkhachroum A Der-Nigoghossian CA Mathews E Massad N Letchinger R Doyle K Ketamine to treat super-refractory status epilepticus. Neurology. (2020) 95(16):e2286–94. 10.1212/WNL.0000000000010611 32873691 PMC7713785

[15] Lagae L. Paediatric Status epilepticus: finally, some evidence-based treatment guidance, but still a long way to go. Lancet Child Adolesc Health. (2020) 4(5):351–2. 10.1016/S2352-4642(20)30030-4 32336347

[16] Agarwal M Fox SM. Pediatric seizures. Emerg Med Clin North Am. (2013) 31(3):733–54. 10.1016/j.emc.2013.04.001 23915601

[17] Swarnalingam E Woodward K Esser M Jacobs J. Management and prognosis of pediatric status epilepticus. Z. Epileptol*.* (2022) 35(4):332–44. 10.1007/s10309-022-00538-0

[18] Van Hove JLK Coughlin C II Swanson M Hennermann JB. Nonketotic hyperglycinemia. In: Adam MP Everman DB Mirzaa GM Pagon RA Wallace SE Bean LJ Gripp KW Amemiya A, editors. Genereviews®. Seattle, WA: University of Washington (1993). 20301531

[19] Keros S Buraniqi E Alex B Antonetty A Fialho H Hafeez B Increasing ketamine use for refractory status epilepticus in US pediatric hospitals. J Child Neurol. (2017) 32(7):638–46. 10.1177/0883073817698629 28349774

[20] Korman SH Wexler ID Gutman A Rolland MO Kanno J Kure S. Treatment from birth of nonketotic hyperglycinemia due to a novel GLDC mutation. Ann Neurol 2006, 59 (2), 411–5. 10.1002/ana.20759 16404748

[21] Rostaminejad M Rostaminejad A. Treatment of refractory status epilepticus with intravenous anesthetic agents: a systematic review. Trends Anaesth Crit Care. (2022) 44:8–19. 10.1016/j.tacc.2022.04.003

[22] Jacobwitz M Mulvihill C Kaufman MC Gonzalez AK Resendiz K MacDonald JM Ketamine for management of neonatal and pediatric refractory status epilepticus. Neurology. (2022) 99(12):e1227–38. 10.1212/WNL.0000000000200889 35817569 PMC10499431

[23] Rossi S Daniele I Bastrenta P Mastrangelo M Lista G. Early myoclonic encephalopathy and nonketotic hyperglycinemia. Pediatr Neurol. (2009) 41(5):371–4. 10.1016/j.pediatrneurol.2009.05.005 19818941

[24] Khraim W Abu-Libdeh B Ayesh S Dweikat I. Clinical heterogeneity of glycine encephalopathy in three palestinian siblings: a novel mutation in the glycine decarboxylase (GLDC) gene. Brain Dev. (2017) 39(7):601–5. 10.1016/j.braindev.2017.03.005 28325525

[25] Poothrikovil RP Al Thihli K Al Futaisi A Al Murshidi F. Nonketotic hyperglycinemia: two case reports and review. Neurodiagn J. (2019) 59(3):142–51. 10.1080/21646821.2019.1645549 31433733

[26] Demirel N Bas AY Zenciroglu A Aydemir C Kalkanoglu S Coskun T. Neonatal non-ketotic hyperglycinemia: report of five cases. Pediatr Int. (2008) 50(1):121–3. 10.1111/j.1442-200X.2007.02513.x 18279221

[27] Suzuki Y Kure S Oota M Hino H Fukuda M. Nonketotic hyperglycinemia: proposal of a diagnostic and treatment strategy. Pediatr Neurol. (2010) 43(3):221–4. 10.1016/j.pediatrneurol.2010.04.018 20691948

[28] Atay E Bozaykut A Sezer G. Four cases of neonatal non-ketotic hyperglycinaemia. Ann Trop Paediatr. (2004) 24(4):345–7. 10.1179/027249304225019172 15720892

[29] Rosati A Ilvento L L'Erario M De Masi S Biggeri A Fabbro G Efficacy of ketamine in refractory convulsive status epilepticus in children: a protocol for a sequential design, multicentre, randomised, controlled, open-label, non-profit trial (KETASER01). BMJ Open. (2016) 6(6):e011565. 10.1136/bmjopen-2016-011565 27311915 PMC4916612

[30] Samanta D. Ketamine in refractory neonatal seizures. Pediatr Neurol. (2020) 106:76. 10.1016/j.pediatrneurol.2019.11.012 31917102

[31] Huntsman RJ. Response to samanta “are ketamine infusions a viable therapeutic option for refractory neonatal seizures?”. Pediatr Neurol 2020;106:76–7. 10.1016/j.pediatrneurol.2020.02.001 32173160

[32] Mandal V Andrews A Tirol F Kaushal S Pergami P. 2022 Eastern regional meeting. J Invest Med. (2022) 70(4):977–1202. 10.1136/jim-2022-ERM

[33] Huntsman RJ Strueby L Bingham W. Are ketamine infusions a viable therapeutic option for refractory neonatal seizures? Pediatr Neurol. (2020) 103:8–11. 10.1016/j.pediatrneurol.2019.09.003 31601453

[34] Freibauer A Jones K. KCNQ2 mutation in an infant with encephalopathy of infancy with migrating focal seizures. Epileptic Disord. (2018) 20(6):541–4. 10.1684/epd.2018.1011 30530441

[35] Tarocco A Ballardini E Garani G. Use of ketamine in a newborn with refractory status epilepticus: a case report. Pediatr Neurol. (2014) 51(1):154–6. 10.1016/j.pediatrneurol.2014.03.006 24938144

[36] Dhamija R Mack KJ. A 2-day-old baby girl with encephalopathy and burst suppression on EEG. Nonketotic hyperglycinemia. Neurology 2011, 77 (3), e16–19. 10.1212/WNL.0b013e318225aae3 21768594

[37] Haider N Salih MA Al-Rasheed S Al-Mofada S Krahn PM Kabiraj M. Nonketotic hyperglycinemia: a life-threatening disorder in Saudi newborns. Ann Saudi Med. (1996) 16(4):400–4. 10.5144/0256-4947.1996.400 17372475

[38] Tegtmeyer-Metzdorf H Roth B Günther M Theisohn M Heinemann U Adams HA Ketamine and strychnine treatment of an infant with nonketotic hyperglycinaemia. Eur J Pediatr. (1995) 154(8):649–53. 10.1007/BF02079070 7588967

[39] Glass HC Shellhaas RA Wusthoff CJ Chang T Abend NS Chu CJ Neonatal seizure registry study group. Contemporary profile of seizures in neonates: a prospective cohort study. J Pediatr. (2016) 174:98–103.e1. 10.1016/j.jpeds.2016.03.035 27106855 PMC4925241

[40] Douglas-Escobar M Weiss MD. Hypoxic-ischemic encephalopathy: a review for the clinician. JAMA Pediatr. (2015) 169(4):397–403. 10.1001/jamapediatrics.2014 25685948

[41] Pellegrin S Munoz FM Padula M Heath PT Meller L Top K Brighton collaboration neonatal seizures working group. Neonatal seizures: case definition & guidelines for data collection, analysis, and presentation of immunization safety data. Vaccine. (2019) 37(52):7596–609. 10.1016/j.vaccine.2019.05.031 31783981 PMC6899436

[42] Shellhaas RA Chang T Tsuchida T Scher MS Riviello JJ Abend NS The American clinical neurophysiology society’s guideline on continuous electroencephalography monitoring in neonates. J Clin Neurophysiol. (2011) 28(6):611–7. 10.1097/WNP.0b013e31823e96d7 22146359

[43] Shanker A Abel JH Schamberg G Brown EN. Etiology of burst suppression EEG patterns. Front Psychol. (2021) 12:673529. 10.3389/fpsyg.2021.673529 34177731 PMC8222661

[44] Harris ML Malloy KM Lawson SN Rose RS Buss WF Mietzsch U. Standardized treatment of neonatal status epilepticus improves outcome. J Child Neurol. (2016) 31(14):1546–54. 10.1177/0883073816664670 27581850

[45] Sharpe C Reiner GE Davis SL Nespeca M Gold JJ Rasmussen M Levetiracetam versus phenobarbital for neonatal seizures: a randomized controlled trial. Pediatrics. (2020) 145(6):e20193182. 10.1542/peds.2019-3182 32385134 PMC7263056

[46] Glass HC Kan J Bonifacio SL Ferriero DM. Neonatal seizures: treatment practices among term and preterm infants. Pediatr Neurol. (2012) 46(2):111–5. 10.1016/j.pediatrneurol.2011.11.006 22264706 PMC3266555

[47] Chien YH Hsu CC Huang A Chou SP Lu FL Lee WT Poor outcome for neonatal-type nonketotic hyperglycinemia treated with high-dose sodium benzoate and dextromethorphan. J Child Neurol. (2004) 19(1):39–42. 10.1177/08830738040190010702 15032382

[48] Mastrangelo M Canafoglia L Franceschetti S Oppezzo C Mosca F Menni F High-frequency rhythmic cortical myoclonus in a long-surviving patient with nonketotic hypergylcemia. J Child Neurol. (2008) 23(3):321–4. 10.1177/0883073807308699 18182648

[49] Yamada J Okabe A Toyoda H Kilb W Luhmann HJ Fukuda A. Cl- uptake promoting depolarizing GABA actions in immature rat neocortical neurones is mediated by NKCC1. J Physiol. (2004) 557(Pt 3):829–41. 10.1113/jphysiol.2004.062471 15090604 PMC1665166

[50] Jensen FE. Developmental factors in the pathogenesis of neonatal seizures. J Pediatr Neurol. (2009) 7(1):5–12. 10.3233/JPN-2009-0270 20191097 PMC2828632

[51] Goodkin HP Yeh JL Kapur J. Status epilepticus increases the intracellular accumulation of GABAA receptors. J. Neurosci*.* 2005, 25 (23), 5511–20. 10.1523/JNEUROSCI.0900-05.2005 15944379 PMC2878479

[52] Abend NS Jensen FE Inder TE Volpe JJ. Chapter 12—neonatal seizures. In: Volpe JJ Inder TE, editors. Volpe’s neurology of the newborn (Sixth edn). Elsevier (2018). p. 275–321.e14. doi: 10.1016/B978-0-323-42876-7.00012-0.

[53] Sanchez RM Jensen FE. Maturational aspects of epilepsy mechanisms and consequences for the immature brain. Epilepsia. (2001) 42(5):577–85. 10.1046/j.1528-1157.2001.12000.x 11380563

[54] Zhou C Sun H Klein PM Jensen FE. Neonatal seizures Alter NMDA glutamate receptor GluN2A and 3A subunit expression and function in hippocampal CA1 neurons. Front Cell Neurosci. (2015) 9. 10.3389/fncel.2015.00362 PMC4585040 26441533

[55] Dingledine R Borges K Bowie D Traynelis SF. The glutamate receptor Ion channels. Pharmacol Rev. (1999) 51(1):7–61. 10049997

[56] Henschel O Gipson KE Bordey A. GABAA Receptors, anesthetics and anticonvulsants in brain development. CNS Neurol Disord Drug Targets. (2008) 7(2):211–24. 10.2174/187152708784083812 18537647 PMC2557552

[57] Leinekugel X Khalilov I McLean H Caillard O Gaiarsa JL Ben-Ari Y GABA Is the principal fast-acting excitatory transmitter in the neonatal brain. Adv Neurol. (1999) 79:189–201. 10514814

[58] Owens DF Boyce LH Davis MB Kriegstein AR. Excitatory GABA responses in embryonic and neonatal cortical slices demonstrated by gramicidin perforated-patch recordings and calcium imaging. J Neurosci. (1996) 16(20):6414–23. 10.1523/JNEUROSCI.16-20-06414.1996 8815920 PMC6578913

[59] Rakhade SN Jensen FE. Epileptogenesis in the immature brain: emerging mechanisms. Nat Rev Neurol. (2009) 5(7):380–91. 10.1038/nrneurol.2009.80 19578345 PMC2822660

[60] Burman RJ Rosch RE Wilmshurst JM Sen A Ramantani G Akerman CJ Why won’t it stop? The dynamics of benzodiazepine resistance in Status epilepticus. Nat Rev Neurol. (2022) 18(7):428–41. 10.1038/s41582-022-00664-3 35538233

[61] Glass HC Shellhaas RA. Acute symptomatic seizures in neonates. Semin Pediatr Neurol. (2019) 32:100768. 10.1016/j.spen.2019.08.004 31813514

[62] Raspall-Chaure M Chin RF Neville BG Scott RC. Outcome of paediatric convulsive Status epilepticus: a systematic review. The Lancet Neurology. (2006) 5(9):769–79. 10.1016/S1474-4422(06)70546-4 16914405

[63] Chin RF Neville BG Peckham C Bedford H Wade A Scott RC. NLSTEPSS collaborative group. Incidence, cause, and short-term outcome of convulsive status epilepticus in childhood: prospective population-based study. Lancet. (2006) 368(9531):222–9. 10.1016/S0140-6736(06)69043-0 16844492

[64] Craven R. Ketamine. Anaesthesia. (2007) 62(Suppl 1):48–53. 10.1111/j.1365-2044.2007.05298.x 17937714

[65] Griesdale DE Bosma TL Kurth T Isac G Chittock DR. Complications of endotracheal intubation in the critically ill. Intensive Care Med. (2008) 34(10):1835–42. 10.1007/s00134-008-1205-6 18604519

[66] Schmutzhard E Pfausler B. Complications of the management of status epilepticus in the intensive care unit. Epilepsia (2011) 52(Suppl 8):39–41. 10.1111/j.1528-1167.2011.03233.x 21967359

[67] Carroll CL Spinella PC Corsi JM Stoltz P Zucker AR. Emergent endotracheal intubations in children: be careful if it’s late when you intubate. Pediatr Crit Care Med. (2010) 11(3):343–8. 10.1097/PCC.0b013e3181ce6d19 20464775

[68] Gray T Dass M. Ketamine cystitis: an emerging diagnostic and therapeutic challenge. Br J Hosp Med (Lond) (2012) 73(10):576–9. 10.12968/hmed.2012.73.10.576 23124288

[69] Middela S Pearce I. Ketamine-induced vesicopathy: a literature review. Int J Clin Pract. (2011) 65(1):27–30. 10.1111/j.1742-1241.2010.02502.x 21155941

[70] de Tymowski C Dépret F Dudoignon E Legrand M Mallet V on behalf of the Keta-Cov Research Group. Ketamine-induced cholangiopathy in ARDS patients. Intensive Care Med. (2021) 47(10):1173–4. 10.1007/s00134-021-06482-3 34313797 PMC8315088

[71] Wang JW Kivovich V Gordon L. Ketamine abuse syndrome: hepatobiliary and urinary pathology among adolescents in flushing, NY. Pediatr Emerg Care. (2017) 33(8):e24. 10.1097/PEC.0000000000000502 26247263

[72] Li T Luo Z Liu Y Wang M Yu X Cao C Excessive activation of NMDA receptors induced neurodevelopmental brain damage and cognitive deficits in rats exposed to intrauterine hypoxia. Neurochem Res. (2018) 43(3):566–80. 10.1007/s11064-017-2451-1 29260492

[73] Jevtovic-Todorovic V Hartman RE Izumi Y Benshoff ND Dikranian K Zorumski CF Early exposure to common anesthetic agents causes widespread neurodegeneration in the developing rat brain and persistent learning deficits. J Neurosci. (2003) 23(3):876–82. 10.1523/JNEUROSCI.23-03-00876.2003 12574416 PMC6741934

[74] Brambrink AM Evers AS Avidan MS Farber NB Smith DJ Martin LD Ketamine-induced neuroapoptosis in the fetal and neonatal rhesus macaque brain. Anesthesiology. (2012) 116(2):372–84. 10.1097/ALN.0b013e318242b2cd 22222480 PMC3433282

[75] Wang C Liu F Patterson TA Paule MG Slikker W Jr. Preclinical assessment of ketamine. CNS Neurosci Ther. (2013) 19(6):448–53. 10.1111/cns.12079 23462308 PMC6493590

[76] Pisani F Cerminara C Fusco C Sisti L. Neonatal status epilepticus vs recurrent neonatal seizures: clinical findings and outcome. Neurology. (2007) 69(23):2177–85. 10.1212/01.wnl.0000295674.34193.9e 18056582

[77] Ronen GM Buckley D Penney S Streiner DL. Long-term prognosis in children with neonatal seizures: a population-based study. Neurology 2007 69(19):1816–22. 10.1212/01.wnl.0000279335.85797.2c 17984448

