# Supplementary material for: Efficacy and safety of ketamine for neonatal refractory status epilepticus: case report and systematic review
Source: Front Pediatr. 2023 Jun 2;11:1189478. doi: 10.3389/fped.2023.1189478 (PMC10275409; doi:10.3389/fped.2023.1189478)
Supplement: Supplementary file 1 [file Table1.docx]

| Supplementary Table 1. Main causes of neonatal seizures | | |
| --- | --- | --- |
| **Hypoxic–ischaemic encephalopathy**  **Neonatal stroke**  Haemorrhagic (intraventricular, intraparenchymal, subdural, subarachnoid)  Ischemic  **Intracranial infections**  Bacterial meningitis and/or abscess  Viral meningoencephalitis  **Self-limited epilepsies**  Self-limited neonatal epilepsy  Self-limited neonatal-infantile epilepsy | **Cerebral malformations**  **Electrolyte imbalance**  Hypocalcaemia  Hyponatremia  **Hypoglycaemia**  **Storage disorders**  Gaucher’s disease  Tay-Sachs disease  Neuronal ceroid lipofuscinosis  **Maternal drug withdrawal** | **Inborn errors of metabolism**  Amino acids or organic acids disorders  Mitochondrial disorders  Bilirubin encephalopathy  Pyridoxine deficiency  Biotinidase deficiency  Molybdenum cofactor deficiency  Sulphite oxidase deficiency  GLUT1 deficiency  CDG syndrome |
